# A predictive model for post-thoracoscopic surgery pulmonary complications based on the PBNN algorithm

**DOI:** 10.1038/s41598-024-57700-z

**Published:** 2024-03-25

**Authors:** Cheng-Mao Zhou, Qiong Xue, HuiJuan Li, Jian-Jun Yang, Yu Zhu

**Affiliations:** 1 Big Data and Artificial Intelligence Research Group, Department of Anaesthesiology, Central People’s Hospital of Zhanjiang, Zhanjiang, Guangdong China; 2 Big Data and Artificial Intelligence Research Group, Department of Anesthesiology, Pain and Perioperative Medicine, First Affiliated Hospital of Zhengzhou University, Zhengzhou, Henan China

**Keywords:** LGBM, Machine learning, Deep learning, Prediction, PPCs, Health care, Risk factors

## Abstract

We constructed an early prediction model for postoperative pulmonary complications after thoracoscopic surgery using machine learning and deep learning algorithms. The artificial intelligence prediction models were built in Python, primarily using artificial intelligencealgorithms including both machine learning and deep learning algorithms. Correlation analysis showed that postoperative pulmonary complications were positively correlated with age and surgery duration, and negatively correlated with serum albumin. Using the light gradient boosting machine(LGBM) algorithm, weighted feature engineering revealed that single lung ventilation duration, history of smoking, surgery duration, ASA score, and blood glucose were the main factors associated with postoperative pulmonary complications. Results of artificial intelligence algorithms for predicting pulmonary complications after thoracoscopy in the test group: In terms of accuracy, the two best algorithms were Logistic Regression (0.831) and light gradient boosting machine(0.827); in terms of precision, the two best algorithms were Gradient Boosting (0.75) and light gradient boosting machine (0.742); in terms of recall, the three best algorithms were gaussian naive bayes (0.581), Logistic Regression (0.532), and pruning Bayesian neural network (0.516); in terms of F1 score, the two best algorithms were LogisticRegression (0.589) and pruning Bayesian neural network (0.566); and in terms of Area Under Curve(AUC), the two best algorithms were light gradient boosting machine(0.873) and pruning Bayesian neural network (0.869). The results of this study suggest that pruning Bayesian neural network (PBNN) can be used to assess the possibility of pulmonary complications after thoracoscopy, and to identify high-risk groups prior to surgery.

## Introduction

At present, thoracoscopic technique is widely used in thoracic surgery^1^. Thoracoscopic surgery has a much lower incidence of complications than earlier forms of open surgery^2^. Although there has been great progress in surgical techniques such as thoracoscopic approach and perioperative treatment, the incidence of complications after lobectomy remains high^3,4^. Despite this, pneumonectomy invariably results in impaired lung function as well as further pulmonary complications which have an incidence ranging from 15 to 37%^5^. The incidence of PPCs after thoracoscopic pneumonectomy also remains high, due to one-lung ventilation, nerve injury, lung disease and high concentrations of inhaled oxygen during surgery and stress reaction^6,7^.

Pulmonary complications are the leading cause of perioperative mortality during pneumonectomy^8^. They contribute to prolonged hospital stays, as well as increased ICU admissions and hospital costs^9,10^. Postoperative pulmonary complications (PPCs) may also be a driver of tumor-related deaths^11^. However, targeted measures can alleviate and prevent postoperative pulmonary complications^12^. At present, there are many studies on the prediction of pulmonary complications, but there remains no effective and feasible intelligence prediction research model^11,13,14^. Furthermore, there have been few studies on developing models to predict PPCs after thoracoscopic surgery.

At present, artificial intelligence (AI) such as machine learning plays a significant role in clinical diagnosis and treatment. In recent years, machine learning methods have attracted considerable attention, due to their superiority over traditional methods in predicting patient prognosis in a variety of settings and disease conditions^15^. A significant advantage of machine learning techniques lies in their ability to produce more stable predictions by handling complex nonlinear relationships between predictive variables. For example, a recent study has suggested that a variety of AI algorithms can be used to construct prediction models for difficult intubations^16^. Additionally, it has been demonstrated that intelligent algorithms can be used to predict the likelihood of pulmonary complications after emergency gastrointestinal procedures^17^. Machine learning and deep learning techniques can also predict intraoperative bleeding in patients undergoing hepatectomy^18^.

The aim of this study was to construct an early prediction model for PPCs after thoracoscopic surgery using machine learning and deep learning algorithms.

## Methods

### Study population

In this study, patients who had undergone thoracoscopic surgery according to the public BioStudies medical database were analyzed. A total of 905 patients who had undergone thoracoscopic surgery were included. Exclusion criteria: emergency and trauma patients; age < 18 years; patients with preoperative pulmonary infection and/or pleural effusion; patients who had undergone open thoracotomy or whose surgery had been canceled; patients who had undergone a second operation; and patients whose relevant data were incomplete or missing. This study was approved by the Ethics Committee at the First Affiliated Hospital of Zhengzhou University (2020-KY-130). It was exempted from informed consent because it was a retrospective study.

### Data collection

Demographic and clinical variables were collected from multiple patients who had undergone thoracoscopic surgery. General information included age, sex, American Society of Anesthesiologists (ASA) classification, body mass index (BMI), and history of hypertension, diabetes, stroke, heart disease, chronic obstructive pulmonary disease (COPD), smoking, and alcohol use. Preoperative laboratory tests included white blood cell, red blood cell, and platelet counts. Information on surgery and anesthetic management included surgery duration and single lung ventilation duration. The study focused on PPCs, which, according to the definition of European perioperative clinical outcomes, comprise respiratory infection, respiratory failure, pleural effusion, pulmonary atelectasis, pneumothorax, bronchospasm, and aspiration pneumonia^19,20^. In the event that one of these complications was detected within the first seven days after surgery, it was considered a PPC.

### AI algorithms

The AI prediction models were built in Python, primarily using AI algorithms. These included both machine learning and deep learning algorithms, such as Logistic Regression, Decision Tree, Random Forest, Gradient Boosting Decision Tree-Gradient Boosting, Extreme gradient boosting-XGB, light gradient boosting machine-LGBM, Linear Support Vector-LinearSVC, Multilayer Perceptron Classifier-MLPC, Gaussian naive Bayes-gnb, K-nearest neighbors-knn, AdaBoost-adab, Convolutional Neural Network-CNN, Long Short Term Memory-LSTM, Convolutional Neural Network + Recurrent Neural Networks-CNNRNN, Convolutional Neural Network + Long Short Term Memory-CNNLSTM and Pruning Bayesian neural network-PBNN. The dataset was first divided into training and test groups at a ratio of 7:3. Then the AI algorithms were used to build prediction models for data in the training group with fivefold cross-validation. Next, the model’s performance was verified in the test group. The LGBM algorithm was used to analyze and rank the weights of each variable accounting for the PPC. Person correlation was used to analyze the relationship between the individual variables. The data were then normalized. Any missing data were processed using the SimpleImputer package. The model was evaluated based on its ROC curve, accuracy, precision, F1 score, recall, Matthews correlation coefficient(MCC), Specificity and MSE score.

### General statistical analysis

R was used to conduct general analysis. The count data were expressed as percentages, with a χ2 test for group comparisons. Any measurement data conforming to a normal distribution were expressed as ${\overline{\text{x}}} \pm {\text{s}}$, with a *t*-test for group comparisons. A *P* value less than 0.05 was considered significant.

### Ethics approval and consent to participate

This study was approved by the Ethics Committee of the First Affiliated Hospital of Zhengzhou University (2020-KY-130), which exempted the informed consent form because it was a retrospective study.

## Results

### General information

A total of 905 post-thoracoscopic patients were included in this study. In neither the training nor the test groups was there any statistical difference in BMI between the PPC and non-PPC groups (Table 1).

**Table 1:** Patient basic characteristic information.

PPCS	No	Yes	*P* value	No	Yes	*P* value
N	488	145		210	62	
Age (years)	54.4 ± 16.2	65.4 ± 10.7	< 0.001	56.1 ± 13.6	66.7 ± 11.3	< 0.001
MAP (mmHg)	101.3 ± 13.5	102.0 ± 12.5	0.694	102.5 ± 14.3	107.1 ± 15.7	0.031
Duration of operation	163.1 ± 98.0	255.7 ± 116.9	< 0.001	151.7 ± 79.9	275.2 ± 120.3	< 0.001
Duration_of one lung ventilation	99.2 ± 58.6	130.8 ± 54.0	< 0.001	98.4 ± 51.8	140.7 ± 57.1	< 0.001
BMI (kg/m^2^)	22.2 ± 3.6	22.1 ± 3.3	0.635	22.7 ± 3.7	23.1 ± 3.4	0.493
Leukocyte counts (× 10^9^/L)	6.1 ± 2.2	6.2 ± 2.6	0.849	5.9 ± 1.7	6.0 ± 2.6	0.732
Red blood cell counts (× 10^12^/L)	4.4 ± 0.6	4.3 ± 0.6	0.155	4.4 ± 0.5	4.2 ± 0.6	0.047
Platelet counts (× 10^9^/L)	204.1 ± 61.9	196.3 ± 71.5	0.065	203.0 ± 66.6	186.4 ± 67.2	0.086
Aspartate aminotransferase (U/L)	20.9 ± 17.3	19.6 ± 14.4	0.336	20.7 ± 17.8	20.1 ± 16.1	0.800
Alanine aminotransferase (U/L)	22.1 ± 10.3	21.6 ± 8.0	0.586	21.9 ± 8.7	23.5 ± 13.0	0.282
Blood glucose (mmol/L)	5.3 ± 1.1	5.4 ± 1.4	0.656	5.4 ± 1.2	5.3 ± 1.3	0.879
Blood creatinine (µmol/L)	64.1 ± 17.0	67.8 ± 21.1	0.065	63.6 ± 19.9	68.5 ± 17.9	0.080
Blood urea nitrogen (mmol/L)	5.5 ± 1.8	5.7 ± 1.8	0.397	5.6 ± 1.4	5.8 ± 2.1	0.233
Serum sodium (mmol/L)	141.2 ± 6.4	141.9 ± 2.6	0.032	141.7 ± 2.0	141.9 ± 2.2	0.376
Serum potassium (mmol/L)	4.1 ± 0.4	4.0 ± 0.4	0.259	4.0 ± 0.4	4.0 ± 0.4	0.479
Serum albumin (g/L)	40.2 ± 4.1	38.9 ± 4.2	0.005	39.8 ± 4.2	37.8 ± 5.5	0.002
Sex			0.051			0.003
Female	188 (38.5%)	43 (29.7%)		87 (41.4%)	13 (21.0%)	
Male	300 (61.5%)	102 (70.3%)		123 (58.6%)	49 (79.0%)	
ASA			< 0.001			< 0.001
1	40 (8.2%)	1 (0.7%)		10 (4.8%)	0 (0.0%)	
2	419 (85.9%)	67 (46.2%)		182 (86.7%)	35 (56.5%)	
3	29 (5.9%)	75 (51.7%)		18 (8.6%)	27 (43.5%)	
4	0 (0.0%)	2 (1.4%)		0 (0.0%)	0 (0.0%)	
Airway management			< 0.001			< 0.001
Single-lumen	18 (3.7%)	1 (0.7%)		6 (2.9%)	4 (6.5%)	
Double-lumen	296 (60.7%)	71 (49.0%)		147 (70.0%)	25 (40.3%)	
Bronchial occluder	165 (33.8%)	73 (50.3%)		57 (27.1%)	33 (53.2%)	
LMA	9 (1.8%)	0 (0.0%)				
History of hypertension			0.021			0.688
No	379 (77.7%)	99 (68.3%)		161 (76.7%)	46 (74.2%)	
Yes	109 (22.3%)	46 (31.7%)		49 (23.3%)	16 (25.8%)	
History of diabetes mellitus			0.005			0.710
No	465 (95.3%)	129 (89.0%)		201 (95.7%)	60 (96.8%)	
Yes	23 (4.7%)	16 (11.0%)		9 (4.3%)	2 (3.2%)	
History of stroke			< 0.001			0.115
No	470 (96.3%)	126 (86.9%)		205 (97.6%)	58 (93.5%)	
Yes	18 (3.7%)	19 (13.1%)		5 (2.4%)	4 (6.5%)	
History of COPD			< 0.001			
No	481 (98.6%)	116 (80.0%)		202 (96.2%)	58 (93.5%)	0.373
Yes	7 (1.4%)	29 (20.0%)		8 (3.8%)	4 (6.5%)	
History of smoking			< 0.001			< 0.001
No	416 (85.2%)	68 (46.9%)		171 (81.4%)	32 (51.6%)	
Yes	72 (14.8%)	77 (53.1%)		39 (18.6%)	30 (48.4%)	

### Correlation analysis between clinical PPC variables

**Figure 1:**
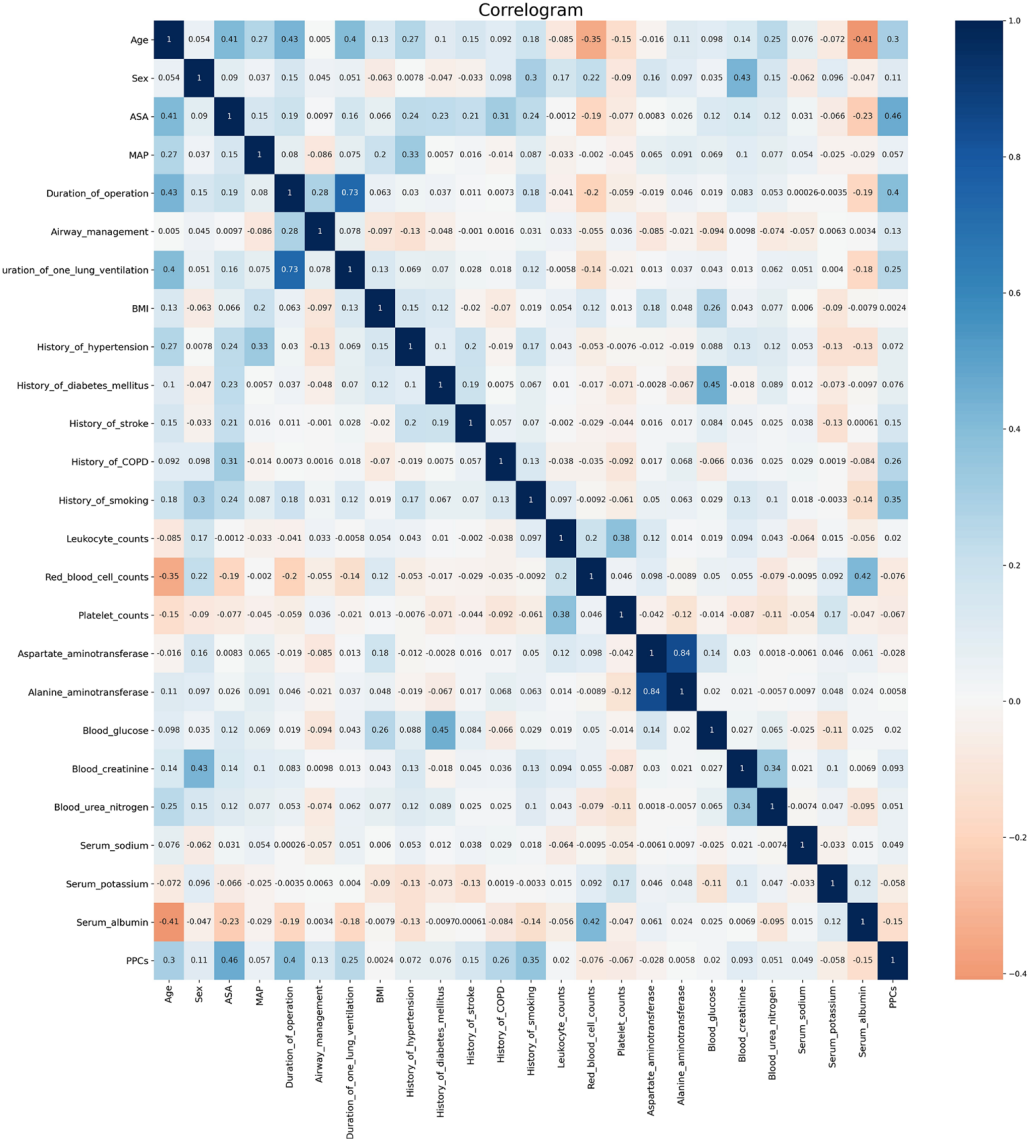
Correlation between variables.

**Figure 2:**
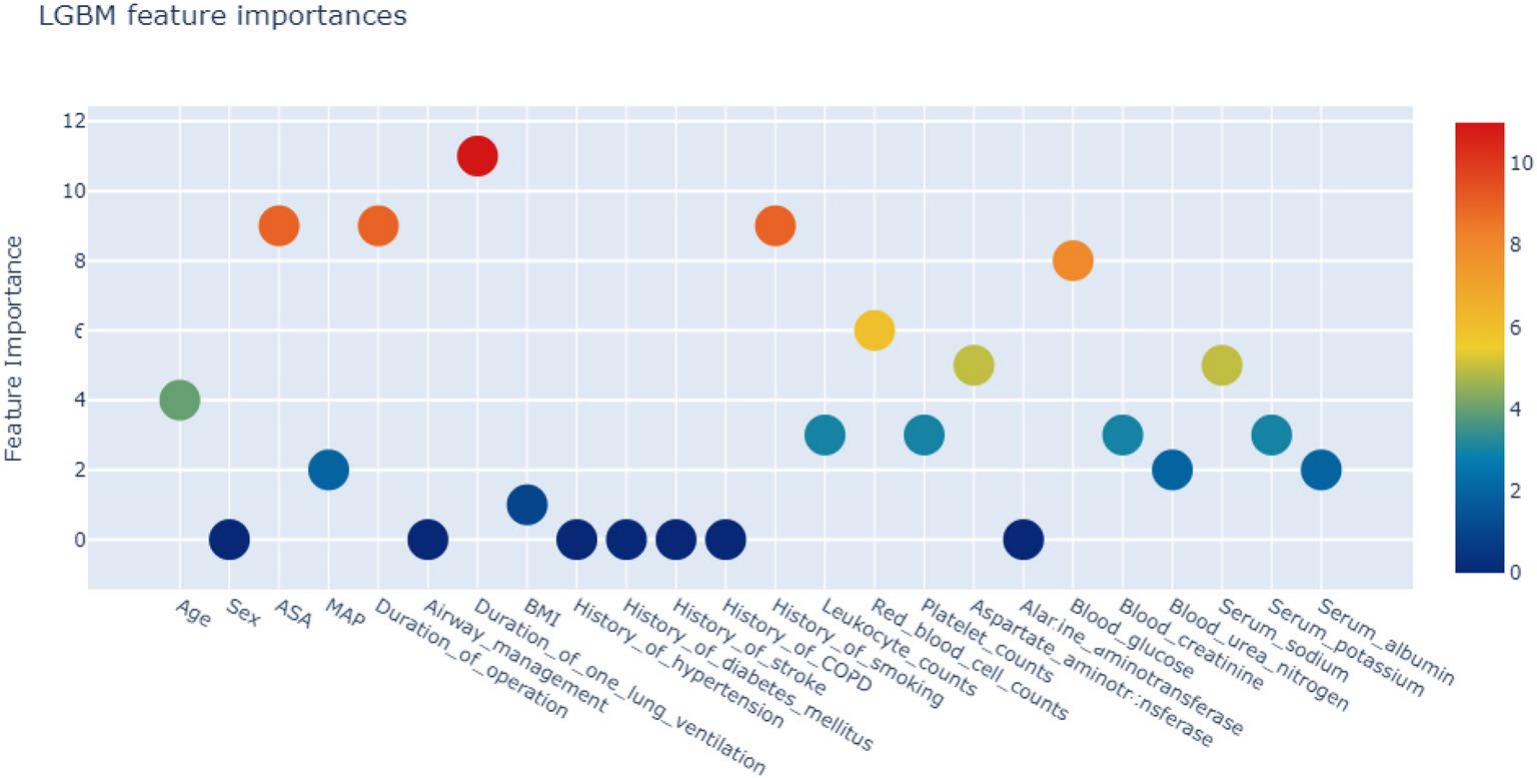
Variable importance of features included in machine learning algorithm.

Correlation analysis showed that PPCs were positively correlated with age and surgery duration, and negatively correlated with serum albumin (Fig. 1). Using the LGBM algorithm, weighted feature engineering revealed that single lung ventilation duration, history of smoking, surgery duration, ASA score, and blood glucose were the main factors associated with PPCs (Fig. 2).

### Results of AI algorithms for predicting pulmonary complications after thoracoscopy in the training group

**Figure 3:**
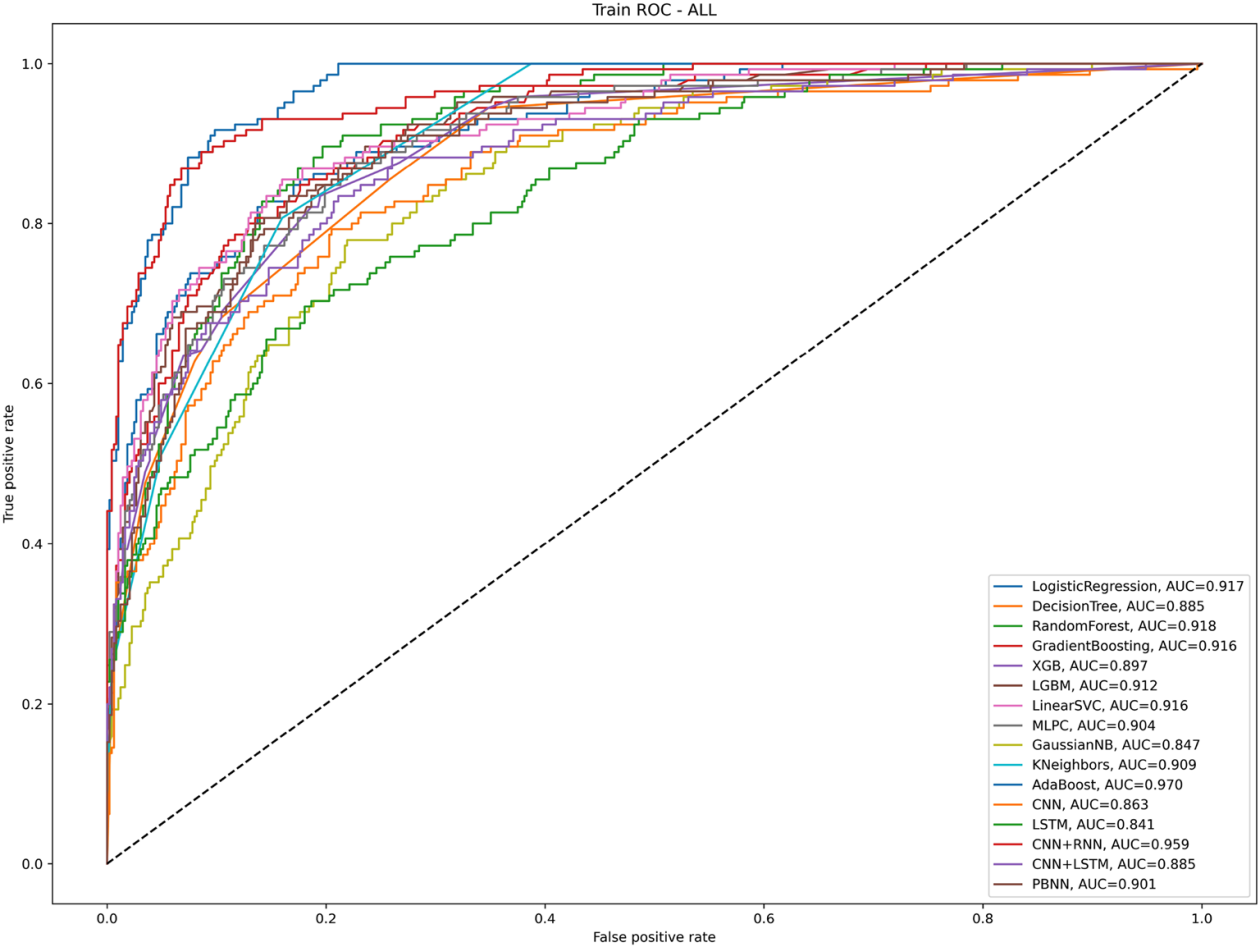
Different AI algorithms predict the PPCs in the training group. Abbreviate: Logistic Regression, Decision Tree, Random Forest, Gradient Boosting Decision Tree-Gradient Boosting, Extreme gradient boosting-XGB, light gradient boosting machine-LGBM, Linear Support Vector-LinearSVC, Multilayer Perceptron Classifier-MLPC, Gaussian naive Bayes-gnb, K-nearst neighbors-knn, AdaBoost-adab, Convolutional Neural Network-CNN, Long Short Term Memory-LSTM, Convolutional Neural Network + Recurrent Neural Networks-CNNRNN, Convolutional Neural Network + Long Short Term Memory-CNNLSTM and Pruning Bayesian neural network-PBNN.

In terms of accuracy, the two best algorithms were adab (0.915) and CNNRNN (0.912); in terms of precision, the best algorithm was RandomForest (0.944); in terms of recall, the two best algorithms were CNNRNN (0.752) and adab (0.745); in terms of F1 score, the best algorithm was CNNRNN (0.796); and in terms of AUC, the two best algorithms were CNNRNN (0.959) and RandomForest (0.918); MCC value greater than 0.6 includes the following algorithms: CNNRNN, adab, logistic regression, linear SVC, and PBNN; and except for the gnb algorithm, the specificity values of other algorithms are all greater than 0.900 (Table 2 and Fig. 3).

**Table 2:** Forecast results for training group.

Model name	AUC	Accuracy	Precision	Recall	F1	Mse	MCC	Specificity
Logistic regression	0.917	0.886	0.807	0.662	0.727	0.114	0.661	0.953
Decision tree classifier	0.885	0.853	0.802	0.476	0.597	0.147	0.541	0.965
Random forest classifier	0.918	0.821	0.944	0.234	0.376	0.179	0.418	0.996
Gradient boosting classifier	0.916	0.848	0.877	0.393	0.543	0.152	0.522	0.984
XGB classifier	0.897	0.858	0.789	0.517	0.625	0.142	0.515	0.977
LGBM classifier	0.912	0.858	0.867	0.448	0.591	0.142	0.556	0.980
LinearSVC	0.916	0.877	0.813	0.600	0.690	0.123	0.627	0.959
MLPC	0.904	0.864	0.847	0.497	0.626	0.136	0.579	0.973
gnb	0.847	0.806	0.568	0.634	0.599	0.194	0.473	0.857
knn	0.909	0.850	0.755	0.510	0.609	0.150	0.536	0.951
adab	0.970	0.915	0.864	0.745	0.800	0.085	0.749	0.965
CNN	0.863	0.831	0.694	0.469	0.56	0.169	0.502	0.941
LSTM	0.841	0.834	0.744	0.421	0.537	0.166	0.459	0.934
CNNRNN	0.959	0.912	0.845	0.752	0.796	0.088	0.821	0.973
CNNLSTM	0.885	0.863	0.790	0.545	0.645	0.137	0.589	0.930
PBNN	0.901	0.853	0.686	0.662	0.674	0.147	0.611	0.959

Abbreviate: Logistic Regression, Decision Tree, Random Forest, Gradient Boosting Decision Tree-Gradient Boosting, Extreme gradient boosting-XGB, light gradient boosting machine-LGBM, Linear Support Vector-LinearSVC, Multilayer Perceptron Classifier-MLPC, Gaussian naive Bayes-gnb, K-nearst neighbors-knn, AdaBoost-adab, Convolutional Neural Network-CNN, Long Short Term Memory-LSTM , Convolutional Neural Network + Recurrent Neural Networks-CNNRNN, Convolutional Neural Network + Long Short Term Memory-CNNLSTM and Pruning Bayesian neural network-PBNN; Matthews correlation coefficient-MCC.

### Results of AI algorithms for predicting pulmonary complications after thoracoscopy in the test group

**Figure 4:**
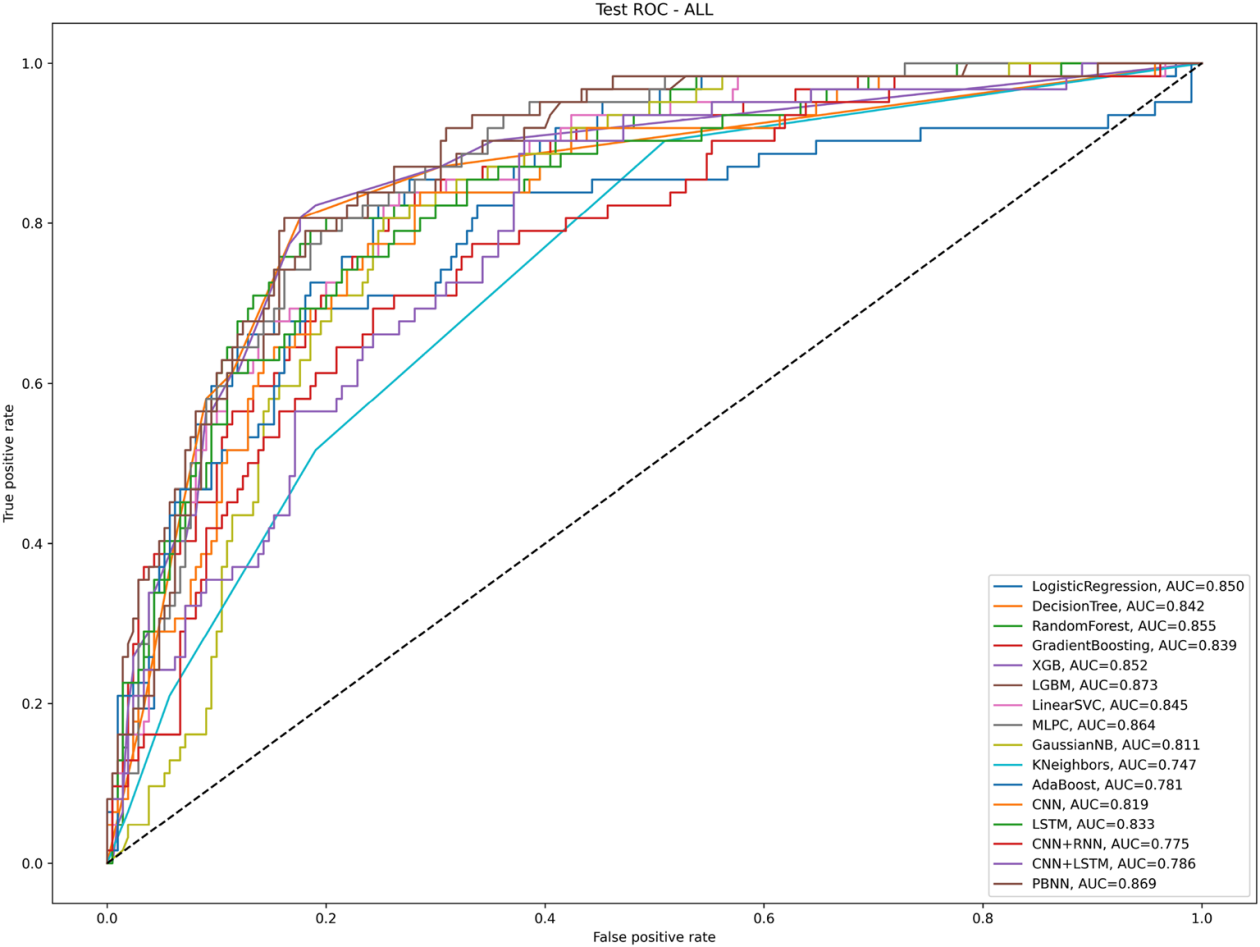
Different AI algorithms predict the PPCs in the test group. Abbreviate: Logistic Regression, Decision Tree, Random Forest, Gradient Boosting Decision Tree-Gradient Boosting, Extreme gradient boosting-XGB, light gradient boosting machine-LGBM, Linear Support Vector-LinearSVC, Multilayer Perceptron Classifier-MLPC, Gaussian naive Bayes-gnb, K-nearst neighbors-knn, AdaBoost-adab, Convolutional Neural Network-CNN, Long Short Term Memory-LSTM, Convolutional Neural Network + Recurrent Neural Networks-CNNRNN, Convolutional Neural Network + Long Short Term Memory-CNNLSTM and Pruning Bayesian neural network-PBNN.

In terms of accuracy, the two best algorithms were LogisticRegression (0.831) and LGBM (0.827); in terms of precision, the two best algorithms were GradientBoosting (0.75) and LGBM (0.742); in terms of recall, the three best algorithms were gnb (0.581), LogisticRegression (0.532), and PBNN (0.516); in terms of F1 score, the two best algorithms were LogisticRegression (0.589) and PBNN (0.566); and in terms of AUC, the two best algorithms were LGBM (0.873) and PBNN (0.869); the algorithms with the highest MCC value were logistic regression and PBNN; and except for the CNNLSTM, CNNRNN, and gnb algorithms, the specificity values of other algorithms are all greater than 0.900 (Table 3 and Fig. 4).

**Table 3:** Forecast results for testing group.

Model name	AUC	Accuracy	Precision	Recall	F1	Mse	MCC	Specificity
Logistic regression	0.850	0.831	0.660	0.532	0.589	0.169	0.489	0.919
Decision tree classifier	0.842	0.816	0.658	0.403	0.500	0.184	0.413	0.938
Random forest classifier	0.855	0.779	0.625	0.081	0.143	0.221	0.165	0.986
Gradient boosting classifier	0.839	0.809	0.750	0.242	0.366	0.191	0.351	0.976
XGB classifier	0.852	0.809	0.625	0.403	0.490	0.191	0.435	0.971
LGBM classifier	0.873	0.827	0.742	0.371	0.495	0.173	0.439	0.962
LinearSVC	0.845	0.816	0.667	0.387	0.490	0.184	0.408	0.943
MLPC	0.864	0.801	0.618	0.339	0.438	0.199	0.351	0.938
gnb	0.811	0.787	0.529	0.581	0.554	0.213	0.415	0.848
knn	0.747	0.776	0.520	0.21	0.299	0.224	0.221	0.943
adab	0.781	0.816	0.630	0.468	0.537	0.184	0.433	0.919
CNN	0.819	0.794	0.571	0.387	0.462	0.206	0.334	0.914
LSTM	0.833	0.809	0.647	0.355	0.458	0.191	0.418	0.933
CNNRNN	0.775	0.783	0.528	0.452	0.487	0.217	0.345	0.857
CNNLSTM	0.786	0.787	0.556	0.323	0.408	0.213	0.308	0.871
PBNN	0.869	0.820	0.627	0.516	0.566	0.180	0.452	0.914

Abbreviate: Logistic Regression, Decision Tree, Random Forest, Gradient Boosting Decision Tree-Gradient Boosting, Extreme gradient boosting-XGB, light gradient boosting machine-LGBM, Linear Support Vector-Linear SVC, Multilayer Perceptron Classifier-MLPC, Gaussian naive Bayes-gnb, K-nearst neighbors-knn, AdaBoost-adab, Convolutional Neural Network-CNN, Long Short Term Memory-LSTM, Convolutional Neural Network + Recurrent Neural Networks-CNNRNN, Convolutional Neural Network + Long Short Term Memory-CNNLSTM and Pruning Bayesian neural network-PBNN; Matthews correlation coefficient-MCC.

Taken together, PBNN performed best among these AI algorithms in predicting post-thoracoscopic pulmonary complications.

## Discussion

With its high incidence, PPCs associated with pneumonectomy are a major contributor to prolonged hospitalization, increased postoperative mortality, and medical costs^21,22^. In spite of the use of perioperative pulmonary protective ventilation strategies and minimally invasive thoracoscopic techniques, the incidence of PPCs remains between 12 and 50%^23^. As a result, preventing PPCs is crucial to the prognosis of post-thoracoscopic patients. In this study, PBNN were found to outperform other AI algorithms in predicting PPCs.

The weighted feature engineering constructed by the LGBM algorithm indicated that the main factors for developing pulmonary complications after thoracoscopy were single-lung ventilation duration, smoking history, surgery duration, ASA score, and blood glucose. The occurrence of PPCs has been shown to be closely related to preoperative interstitial pneumonia and smoking history^24^. A predictive risk model for PPCs can be constructed using age, smoking status, and postoperative 1-s forced expiratory volume^25^. PPCs are also associated with prolonged surgery times^26^. In multivariate analysis, the risk factors associated with increased prevalence of PPCs were ASA physical status ≥ III and surgery duration > 5 h^27^. Surgery duration, one-lung ventilation duration, and ASA score are significant predictors of PPCs after thoracic surgery^28^. The incidence of PPCs was 10.9% among the 6,063 patients who were analyzed, and factors such as advanced age, ASA score, and surgery duration ≥ 1 h were the main determinants of pulmonary complications^29^. PPCs have been reported to be significantly influenced by smoking, postoperative blood glucose, and ventilation duration in patients undergoing noncardiac surgery^30^. Additionally, diabetic patients have a higher risk of pulmonary complications during the perioperative period of coronary artery bypass surgery than do non-diabetics^31^. These conclusions are also supported by our findings.

This study does have its limitations. Firstly, it was a retrospective study conducted at a single center, and therefore it is subject to single-center bias. For internal validation, cross-validation were used; however, further multicenter and prospective studies are needed. Furthermore, this retrospective study did not include detailed information on intraoperative hemodynamic fluctuations, postoperative pain, or its treatment.

This study’s results suggest that AI algorithms such as PBNN can be used to assess the possibility of pulmonary complications after thoracoscopy, and to identify high-risk groups prior to surgery. Moreover, PBNN’s accuracy rate is 82%, the AUC value is 0.869, and its recall rate and F1 value are both greater than 0.5. Therefore, AI algorithms should be able to facilitate early intervention which will reduce the likelihood of pulmonary complications, and facilitate the management of patients in the perioperative period.

## Data Availability

The data is available from the BioStudies public database (https://www.ebi.ac.uk/biostudies/europepmc/studies/S-EPMC8572520).
